# Methodical Approach for Determining the Length of Drill Channels in Osteosynthesis

**DOI:** 10.3390/s19163532

**Published:** 2019-08-13

**Authors:** Michael Sorg, Jan Osmers, Andreas Fischer

**Affiliations:** Bremen Institute for Metrology, Automation and Quality Science, University of Bremen, 28359 Bremen, Germany

**Keywords:** handheld bone drilling, sensory drivetrain, orthopedics, linear oscillating shaft

## Abstract

In order to fix a fracture in osteosynthesis, it is necessary to attach screws bicortically to the bone. The length of the screws must be selected correctly in 1-mm increments: otherwise, injury to the surrounding tissue structure or insufficient fixation will result. The drill channel length can only be determined preoperatively to a limited extent and with insufficient accuracy and is therefore determined intraoperatively with a mechanical caliper gauge. This length determination is error-prone, which often leads to a false screw selection and at the same time to considerable complications in the healing process. A novel approach based on a sensory drive train was pursued, with which all mechanical drilling parameters were recorded and evaluated in combination with a length measurement that allows for determining the drill channel length. In order to overcome the limitations of previous drill concepts, a precise length measurement of the drill channel was introduced. The amplitude of a stimulated linear oscillation of the drill was monitored for drilling channel length measurements in order to reliably detect the beginning of the drilling process. The method provides the information required for handheld drilling without the limitation of constant drilling parameters. With initial results from laboratory tests with pig bones, the measurement method for the drill channel length has been validated on a test bench of the drilling machine. With the laboratory tests, a measurement uncertainty of 0.3 mm was achieved, so screws with a 1-mm step width can be reliably selected.

## 1. Introduction

Osteosynthesis is an established method of treating bone fractures. The bone fragments are fixed to a metal plate with the aid of screws in order to align the bones during the healing process in the anatomically correct position [1]. For this purpose, a medical drill is used to drill through the bone. At present, the length of the drill channel is determined manually with a caliper gauge [2]. The appropriate screw length is then selected and inserted into the bone. The false selected screw length can then be checked by X-ray or ultrasound, but both methods have their disadvantages, e.g., exposure to radiation and extension of the operation time. Ultrasound is considered not precise enough to replace X-rays [2]. 

**Figure 1:**
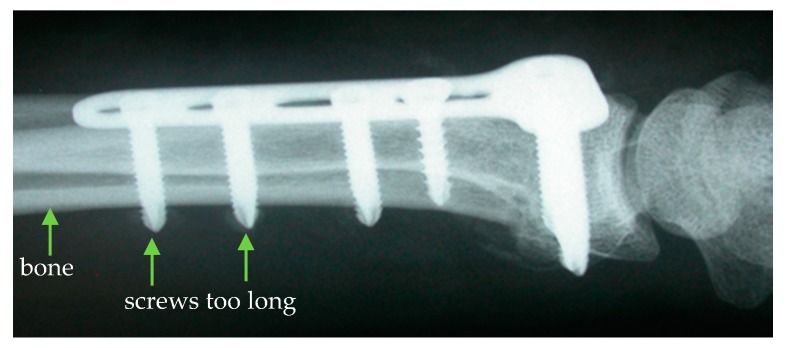
Screw protrusion due to an incorrect determination of the drill channel length [4].

However, the determination of the drill channel length is subject to a high degree of uncertainty, which regularly leads to an incorrect determination of the required screw length. The most common treatment error resulting from this is a screw protrusion in which the screw ends protrude from the bone and can injure adjacent tissue (Figure 1) [3].

In order to avoid incorrect implant placement, it is necessary to measure the drill channel length intraoperatively with a measurement uncertainty that is sufficient for a clear screw selection. The investigated solution was therefore the development of a drilling method in which the length of the drilling channel is measured directly during the drilling process. The aim of the new measurement technique is a resolution of 0.1 mm with a measurement uncertainty of less than 0.5 mm for the drill channel length determination in order to ensure a reliable selection of the screws in 1-mm increments.

Brett et al. [5] published their first strategy for automated breakthrough detection in human ear surgery in 1995. This was based on the realization that at the moment of the drill’s breakthrough, the feed force drops rapidly and the torque increases significantly, provided that the feed rate is kept constant. A comparable breakthrough detection strategy has been presented by Lee et al. 2004 [6], Modh et al. 2012 [7], Hsu et al. 2001 [8] and Ong et al. 1998 [9]. Allotta et al. 1996 [10] proposed the determination of threshold values for the first derivative of the feed force curve as an indicator for breakthrough detection. In 2013, Díaz et al. [11] presented an experimental setup for bone drilling, with which they carried out experiments for automatic breakthrough detection. A force acting on the linear drive when the drill enters hard bone material leads to a difference between the specified reference position and the measured actual position of the linear feed. At the end of the drilling channel, the material provides less resistance, and the deviation between the nominal and the actual position becomes smaller. For mobile use, the authors equipped a portable linear guide unit with a medical drilling tool [11]. Dai et al. 2013 [12] developed an automatic breakthrough detection method especially for use in pedicle drilling, which allows the drill to be stopped before the countercorticalis is broken in order to protect the underlying nerve and blood vessels. The vibrations generated during drilling are evaluated with a wavelet analysis with regard to the specific vibration characteristics of cancellous bone and cortical bone. Guan et al. 2018 [13] pursued a comparable approach by recording the sound emissions during drilling in a frequency-selective manner and evaluating them together with the feed force and torque using an artificial neural network. For robot-assisted drilling and pedicle milling, Hu et al. 2013 [14] used a multiaxis force sensor and a torque sensor to simulate the feeling in a surgeon’s hand when he encounters different bone tissues. The evaluation for reliable breakthrough detection is based on a state recognition model. Osa et al. 2015 [15] presented a first approach to automatic breakthrough detection for hand-held drills by equipping a hand-held drilling and milling machine with a multiaxis acceleration and gyro sensor. In particular, fast changes in the feed motion are evaluated together with the torque, which is indirectly determined via the motor current. This means that the drilling or milling process can be stopped automatically as soon as breakthroughs begin. The solution approaches described in the mentioned literature essentially described interesting procedures for breakthrough detection and thus only described a partial aspect of the determination of the drill channel length, for which the precise detection of the start of the drill channel in combination with a path length measurement is additionally necessary.

The published results for the detection of the breakthrough in bone drilling have mainly been based on progressions of torque and feed force. With the methods presented, however, it is necessary to keep the feed rate [16] or the feed force [11] constant. In addition, the drill must follow an exactly defined path, because these solutions do not directly measure the distance between the drill and the bone. Therefore, recent research has focused on robotic-assisted bone drilling, so that the boundary conditions of a constant feed rate or force are present [17,18,19]. Based on robotic bone drilling, Accini et al. developed algorithms to prevent the drill tip from plunging through the countercortex by comparing the actual feed position to the planned position of the set ramp. A threshold of 3 mm/s was set for the derivative of the position error, successfully preventing the breakthrough in bovine and chicken bones [19]. Sun et al. [18] analyzed the resulting audio signal of the acoustic emissions while drilling in bone and generated a state-recognition method, with recorded frequencies between 8 kHz and 14 kHz. However, with a drill tool guided manually by the operator, the applied feed force and the speed that could be set on the drill tool varied on the basis of experience and feeling. 

For precise measuring of the drill channel length, a new medical drilling method with a sensory drive train is presented, in which all variable drilling parameters are recorded during drilling, which makes the measurement approach suitable for hand-held drilling machines. In Section 2, the measurement approach is presented, where Section 2.1 explains the drilling parameters and Section 2.2 shows the principle of the sensory drive train. In Section 2.3, the laboratory setup of the sensory drive train with a linear guide is described, which was used for drill channel length determination on porcine ribs. In Section 3, the results of the drilling experiments are presented. In Section 4, the experimental results are discussed concerning the observed measurement deviations and the applicability for drill channel length measurements. In Section 5, a conclusion is drawn and a short outlook is given. 

## 2. Materials and Methods

### 2.1. Definition of Drill Parameters

During drilling, the rotary cutting movement of the tool is superimposed with a linear feed movement in the direction of the workpiece. The total power applied for this operation is composed of the cutting power $P_{c}$ and the feed power $P_{f}$:

(1) $$P_{\mathrm{ges}}=P_{c}+P_{f}.$$

The cutting power $P_{c}$ can be calculated as follows (per cutting edge, without losses):

(2) $$P_{c}=2\cdot \pi \cdot n\cdot M,$$

where n is the specified rotational speed, and M is the torque applied by the drive for the rotary movement of the drill at this speed. A basic torque is already required for the rotation of the tool at idling speed, which increases during machining depending on material properties and friction effects. The following applies to the feed power:

(3) $$P_{f}=v_{f}\cdot F_{f},$$

where $v_{f}$ is the feed rate of the tool and $F_{f}$ is the feed force occurring during machining.

The measuring principle is based on the complete measurement of the mechanical quantities describing the drilling process.

In the sensory drive train, the speed *n*, the feed rate $v_{f}$, the torque *M*, and the feed force *F_f_* are therefore recorded and evaluated for detection of the drill breakthrough during bone drilling and to compare it to the achieved feed for determining the drill channel length.

As an additional parameter, the amplitude of a linear oscillation impressed on the drive train is recorded. The experiments investigate whether this additional parameter can improve breakthrough detection in inhomogeneous bone material. 

### 2.2. Sensory Drive Train

The measurement approach is based on a continuous and complete recording of the drilling parameters during drilling, with additional acquisition and evaluation of the linear oscillation of the drill. The combination of the drilling parameters allows for the concept to be transferred to a manually guided drilling machine, where the parameters are varied according to the surgeon’s experience and feeling during drilling. In addition to the mechanical drilling parameters, it is expected that the amplitude of the linear oscillation at material transitions will also vary depending on the material properties such as hardness, density, strength, etc., within an inhomogeneous material layer.

**Figure 2:**
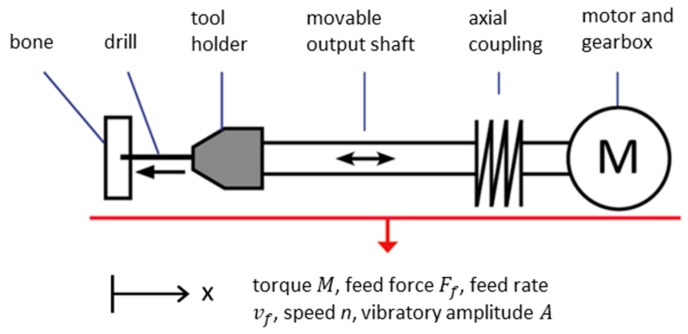
Principle of the sensory drive train.

The technical implementation of the principle is based on a drive train with an axially movable shaft (see Figure 2). A spring coupling enables axial movements in the output shaft relative to the input shaft. The axial mobility of the output shaft allows for easy measurement of the feed force and the use of linear oscillation modulation.

The torque M can be obtained in the sensory drive train from the motor current I. It is in a fixed ratio by a motor-specific torque constant, k:

(4) M=k·I

The motor current can be measured by an electric current sensor.

The feed force $F_{f}$ is determined with the aid of the coupling, which connects the axially movable output shaft with the axially mounted input shaft. The coupling acts like an axial spring, which is compressed under load and extends again when the load is released. The feed force *F_f_* corresponds to the spring force $F_{s}$, which results from the change in length $\Delta l_{fed}$ of the coupling (axial displacement). The ratio between the change in length $\Delta l_{fed}$ and the spring force $F_{s}$ depends on the spring-specific spring constant D:

(5) $$F_{f}=F_{s}=D\cdot \Delta l_{fed}$$

The axial displacement is measured by a distance sensor that is directed from a stationary point of the machine onto the coupling or another point on the moving shaft.

The feed rate $v_{f}$ can be calculated from the derivative of the axial distance *s_lin_* between tool and workpiece over time:

(6) $$v_{f}\left(t\right)=\frac{d}{dt}s_{lin}\left(t\right)$$

The measurement of the distance between tool and workpiece is carried out with an optical distance sensor, which is directed onto the workpiece from the tool.

The speed n of the motor is detected by means of a reflection light sensor by observing a notch rotating with the shaft on the coupling. The reflection light sensor also detects the amplitude A of the linear oscillation on a reference component on the shaft.

In addition to an evaluation of the ratio of energy input into the bore to the drilling progress achieved, the sudden amplitude change of the linear oscillation of the drill bit can thus be evaluated for the detection of the breakthrough through the bone. The length of the drill channel results from the difference in distance between the entry and breakthrough of the drill.

### 2.3. Experimental Setup

**Figure 3:**
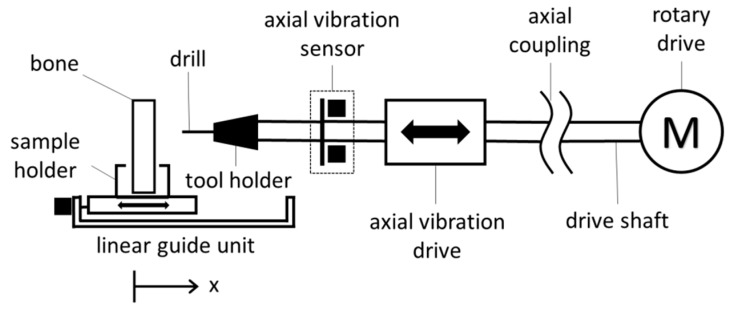
Test rig of the sensory drive train with linear bone feed.

For the characterization and verification of the measuring principle, an experiment was set up in which a pig bone was fed to the drill via a linear guide unit (see Figure 3).

**Figure 4:**
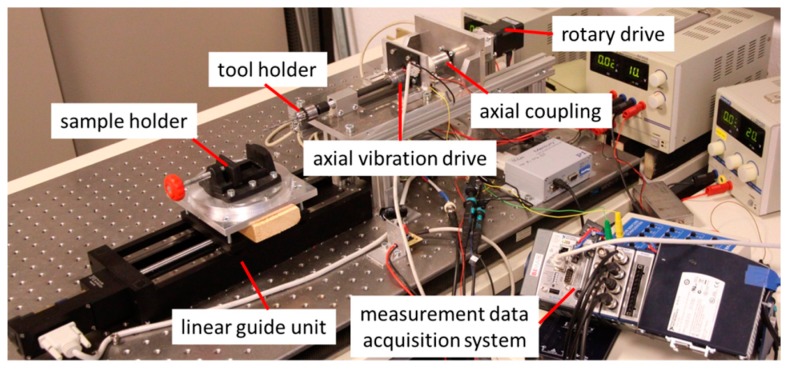
Laboratory setup of the sensory drive train.

The drive train is driven by an electric motor with a speed of 800 rpm and a torque of up to 15 Nm. The rotary movement of the drill is additionally superimposed with a linear oscillation by an axial vibration drive. For this purpose, the drive shaft is connected to the axially vibrating shaft by means of a spring coupling. The axial vibratory drive is realized by an electromagnetic voice coil drive and is supplied with a sinusoidal signal via an audio amplifier. The workpiece is fed with a linear guide unit with a programmable feed rate. Figure 4 shows the laboratory setup of the sensory drive train together with the measurement data acquisition system used.

**Figure 5:**
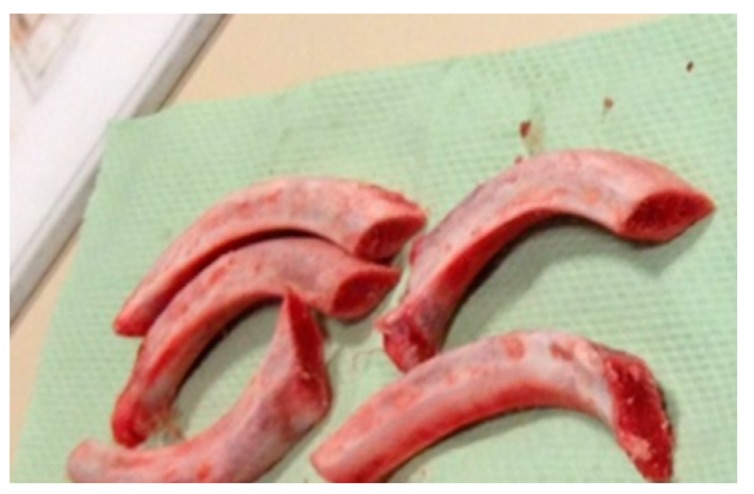
Ribs of the pig.

Fresh porcine ribs were used for the drilling experiments. The attached fat and muscular tissue had previously been removed (see Figure 5).

**Figure 6:**
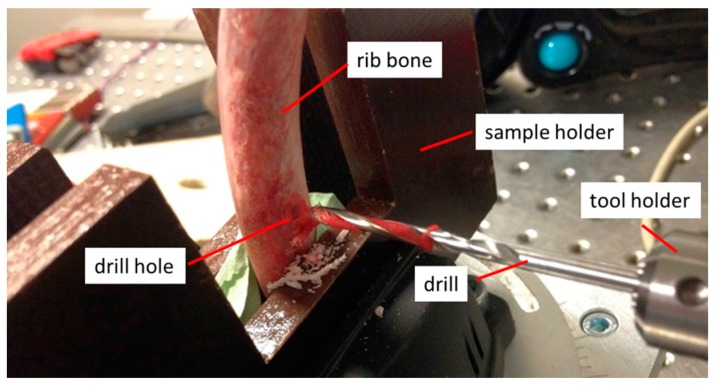
Drilling through a rib bone.

In the rib bones used, the outer bone layers (cortical and countercortical) consisted of compact bone tissue. The internal cancellous bone consisted of a significantly softer sponge-like system of fine bone balls (trabecula). During a drilling process, the drill first penetrates into the cortex and finally reaches the countercortex via the cancellous bone. Figure 6 shows a drilling procedure through a rib bone with a medical twist drill (diameter 3 mm). During drilling, the drill penetrates alternately hard and soft layers. It is therefore expected that the different material properties of the bone layers will individually influence the measured drilling parameters.

Drilling experiments on bone were carried out with a medical twist drill at a constant feed rate of *v_f_* = 0.5 mms^−1^ and a speed of *n* = 600 rpm. The speed and feed rate thus corresponded to typical operational values in osteosynthesis. The axial vibration drive was excited with a sine function with a frequency of 79 Hz. The data were recorded at 5120 Hz and 16 bit with an A/D converter card and were processed with Matlab.

## 3. Results

**Figure 7:**
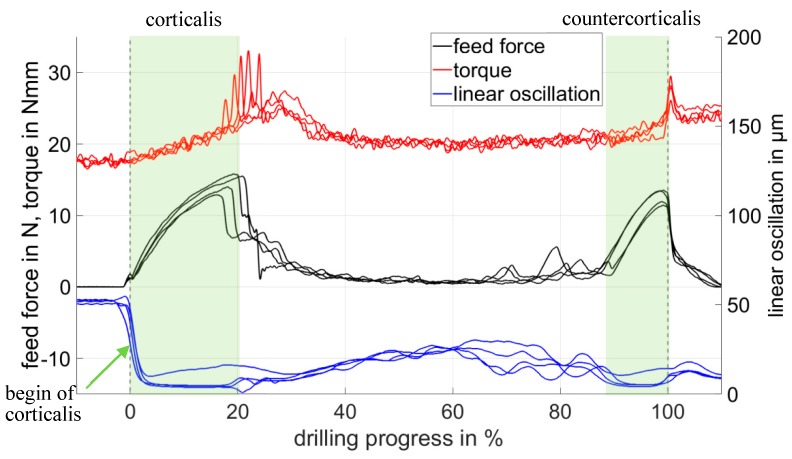
Measuring signals from four rib bores for feed force and linear oscillation measured by the displacement of the spring coupling and torque measured from the motor current.

Figure 7 shows the measured course of motor current (torque), the spring travel of the axial coupling (feed force), and the amplitude of the linear oscillation for four bore experiments with a porcine rib. Due to the different thicknesses of the rib at the bore locations, the drill channel length is normalized and shown as drilling progress in %.

At a drilling progress of 0 mm, the drilling began with contact from the tip of the drill bit on the cortical surface. When penetrating the cortex, the feed force increased, while the linear oscillation amplitude showed a sharp decrease. In addition, the motor current, which was converted into the prevailing torque according to Equation (4), increased. The drill reached the end of the cortex at 2 mm and penetrated the softer cancellous bone. Due to the increased flexibility of the cancellous bone, the feed force was reduced, the spring coupling was partially released again, and the linear oscillation amplitude increased again slightly. When penetrating the countercortex (at 9 mm), the signal curve was similar to that of penetration into the cortex. The breakthrough through the rib occurred at 10 mm and led to a sudden drop in the feed force and to a torque peak. The linear oscillation provides a robust signal with a steeply sloping flank at the beginning of the bone tissue, which is the first required value for the bore channel length calculation.

**Figure 8:**
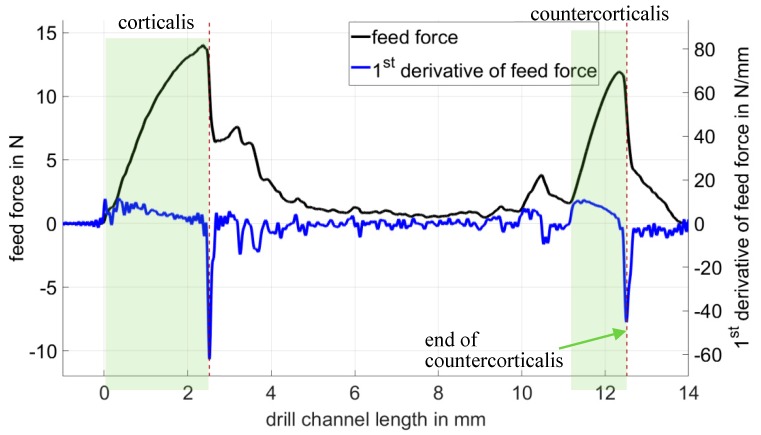
Feed force and its first derivative. The peaks of the derivative show the two breakthroughs of the cortex.

The position of breakthrough was determined by using the first derivative of the feed force (see Figure 8). At the moment of breakthrough through the cortex and through the countercortex of a rib, the first derivative of the feed force showed a minimum due to the steep drop in the feed force. The length of the drill channel resulted from the difference in distance between the breakdown of linear oscillation in Figure 7 and the minimum of the first derivative of the feed force at the moment of the countercorticalis breakthrough in Figure 8. In this way, the drill channel length could be determined in a test series with 10 drill holes through porcine rib bones, with a standard deviation of 0.11 mm and a systematic deviation of 0.1 mm. Since the source of the systematic deviations had not yet been determined, it was treated as a random error in accordance with the “Guide to the expression of uncertainty in measurement”. As a result, the expanded measurement uncertainty (*k_p_* = 2) was 0.31 mm and covered existing systematic and stochastic deviations (see Table 1). The measurement uncertainty of 0.31 mm achieved in the laboratory tests for drill channel length measurement was sufficient to ensure the selection of the correct screw in osteosynthesis treatment. The prominent indicators for the beginning and end of the drill channel were usable for the development of algorithms for automatic length calculation.

## 4. Discussion

The presented measurement curves show the feasibility of the sensory drive train in measuring the drill channel length through evaluation of the linear oscillation breakdown as a robust indicator for the beginning of the bore hole and the first derivative of the feed force as the indicator for the end of the bore hole. The drill channel length results from the difference in the reference position of the drill at the two indication positions. The first results demonstrated that the chosen sensors are capable of achieving a measurement uncertainty in the desired range below 0.5 mm. 

We expected to see a more clear inclination of the linear oscillation when breaking through the cortical bone into the cancellous tissue. Probably due to the friction of the drill in the bore hole and a weak voice coil drive, only the drop of the linear oscillation at bone entry was a useful feature of the measurement concept (so far). The voice coil drive will be designed to be stronger in future measurements to achieve a more clear incline after the breakthrough of cortical bone. It is assumed that the incline after breakthrough will provide another indicator for bone breakthrough that is insensitive to rotational speed and feed rate.

Compared to the approaches from references [6,7,8,9,10,11], the linear oscillation proved to provide further information, especially about the beginning of the drilling process, which clearly indicated the start of the drill channel. Therefore the drill channel length determination became more accurate and robust with the additional information and allowed for the achievement of the presented low measurement uncertainty, without requiring technically complex sound or vibration measurements [12,13].

For the first experiments, variable influences resulting from variations in rotational speed and feed rate that occur in manually guided drilling were kept constant to test the sensor capabilities. As a next step, we expect to include variations in drilling parameters, such as feed rate and rotational speed, to test the robustness of the signal indication points to evaluate the reference distance between entry and breakthrough of the bone. Future research will address the development of algorithms for automatic drill channel length determinations under variations in drill parameters that mimic manually guided drilling.

## 5. Conclusions

With the aim of replacing the error-prone caliper gauge in osteosynthesis in order to enable the selection of a correctly dimensioned screw, a basically in-process capable measuring approach to determine the drill channel length with a sensory drive train has been presented. First, drilling experiments under laboratory conditions on porcine rib bones showed the potential of the sensory drive train method with a total measurement uncertainty of 0.31 mm (*k_p_* = 2). This meets the requirement for a measurement uncertainty of less than 0.5 mm so that a reliable selection of screws available in 1-mm increments can be guaranteed. Further work will examine the measuring concept under varying boundary conditions as they occur with a hand-held bore.

## Figures and Tables

**Table 1:** Overview of measurements on porcine ribs. The reference value was determined with a caliper gauge with an uncertainty of 0.02.

No.	Reference Value (in mm)	Drill Channel Length (in mm)	Deviation (in mm)
1	12.64	12.846	0.206
2	12.58	12.637	0.057
3	11.50	11.467	−0.033
4	11.88	11.855	−0.025
5	9.65	9.683	0.033
6	10.00	10.000	0.000
7	10.40	10.655	0.255
8	11.53	11.793	0.263
9	10.59	10.752	0.162
10	9.20	9.313	0.113
	mean		0.103
	standard deviation		0.1133
expanded measurement uncertainty (*k_p_* = 2)			0.3066
